# Malnutrition is Common in Patients Utilizing Glucagon-Like Peptide-1 Agonists Prior to Total Joint Arthroplasty

**DOI:** 10.1016/j.artd.2025.101865

**Published:** 2025-09-30

**Authors:** Zachary Jodoin, William H. Young, Daanish Sheikh, Belinda Pena, Chance C. Moore, Frank Buttacavoli

**Affiliations:** UT Health Science Center San Antonio, Department of Orthopaedics, San Antonio, TX, USA

**Keywords:** Malnutrition, GLP-1 agonists, Optimization, Rapid recovery, Primary arthroplasty, Weight loss

## Abstract

**Background:**

The rising prevalence of obesity and the increased use of glucagon-like-peptide-1 (GLP-1) receptor agonists for weight loss and diabetes has led to more patients qualifying for elective total joint arthroplasty (TJA). While these medications promote weight reduction, they may predispose patients to nutritional deficiencies. This study aims to evaluate the preoperative nutritional status of GLP-1 users undergoing TJA compared to nonusers.

**Methods:**

A retrospective chart review was conducted at a high-volume center on patients who underwent elective primary TJA between January 1 and May 1, 2025. Patients were included if they had complete preoperative nutritional labs. Nutritional markers included hemoglobin, albumin, total protein, prealbumin, calcium, alkaline phosphatase, and 25-hydroxy vitamin D. Malnutrition was defined as ≥1 laboratory deficiency; severe malnutrition as ≥2 deficiencies. GLP-1 use, indication, and duration were recorded. Statistical analyses included t-tests, chi-square tests, and odds ratio calculations.

**Results:**

A total of 165 patients met inclusion criteria, with 29 (17.6%) actively using GLP-1 receptor agonists. The cohorts were matched for comorbidities. GLP-1 users had higher rates of malnutrition (38% vs 8.8%, *P* < .001; odds ratio = 6.2), severe malnutrition (17.2% vs 2.9%, *P* = .009; odds ratio = 6.88), and lower albumin (*P* < .001), prealbumin (*P* = .003), and total protein (*P* = .024) levels compared to controls.

**Conclusions:**

GLP-1 agonist use is associated with significantly higher rates of preoperative nutritional deficiencies in patients undergoing elective TJA. Given the high risk of malnutrition in this growing patient population, targeted nutritional screening and optimization should be considered standard practice in the preoperative evaluation of GLP-1 users.

## Introduction

The negative impact of obesity on arthroplasty outcomes has been extensively investigated within the orthopaedic literature [[1], [2], [3]]. With the United States obesity rate climbing higher than 42% and the projected increase of arthroplasty surgeries required in the coming years, orthopaedic surgeons and patients alike are searching for new weight loss strategies, including the use of glucagon-like peptide-1 (GLP-1) receptor agonists [4].

GLP-1 agonists have been used for the treatment of type 2 diabetes for decades [5,6]. In recent years, a trend has developed toward the use of these medications to help increase weight loss. These drugs have demonstrated significant efficacy in promoting weight loss by enhancing insulin secretion, reducing glucagon levels, and suppressing appetite. Many studies, including randomized clinical control trials, have shown both larger and more sustained weight loss with the use of GLP-1 inhibitors [[7], [8], [9]]. As a result, within the United States alone, there has been an over 700% increase of patients without diabetes utilizing these medications [10]. Although obesity is a common barrier to safe TJA, malnutrition is also a significant risk factor for surgical complication. This malnutrition risk is well documented in the setting of recent bariatric surgery weight loss [[11], [12], [13], [14], [15]]. No literature to date has identified the nutritional state of patients utilizing GLP-1s prior to TJA. Markers such as prealbumin are commonly utilized for identifying subclinical malnutrition, which may impair wound healing, immune function, and overall recovery from illness or surgery [16]. Prior studies have focused on prescreening and identifying malnutrition in total joint arthroplasty (TJA) patients, identifying an approximately 8.5% malnutrition rate among this unique population [11,[17], [18], [19]].

Despite these malnutrition risks, no study to date has investigated baseline nutritional values for this GLP-1 population. This study aims to identify if this population is at risk for nutritional deficiencies. By assessing these values, it can help guide preoperative monitoring and nutritional optimization for these patients prior to undergoing elective TJA and potentially improve outcomes.

## Material and methods

Following institutional review board exemption, a retrospective chart review was performed at a high-volume academic urban medical center to evaluate the nutritional status of patients undergoing primary TJA with a focus on patients utilizing GLP-1 agonist medication for diabetes, weight loss, or both. The study included all patients who underwent elective primary total knee arthroplasty (TKA) or total hip arthroplasty (THA) between January 1, 2025, to May 1, 2025, and who had complete institution standard nutrition preoperative lab data. Exclusion criteria included any patient undergoing revision or conversion TJA. All patients were required to have a body mass index (BMI) of <45.0, hemoglobin A1c (HbA1c) of 7.5 or less, a hemoglobin value of >10.0, and were required to be nicotine free at the final preoperative evaluation.

Patient demographic data were extracted including procedure type (TKA vs THA), laterality, patient age, race, gender, and BMI. Comorbidity data included a detailed past medical history including hypertension, diabetes mellitus and end organ damage, hyperlipidemia, coronary artery disease, endocrine pathology, prior myocardial infarction, congestive heart failure, peripheral vascular disease, history of stroke, dementia, chronic obstructive pulmonary disease, kidney disease, liver disease, history of cancer or solid tumor, current or past smoking, drug use, and alcohol use. These data were used to calculate a Charlson Comorbidity Index (CCI) for each patient to quantify overall medical burden. Individual comorbidity prevalence was not statistically analyzed between groups, but instead a statistical analysis of the CCI for each group was used as a marker to assess the baseline health of the 2 patient populations. A subgroup analysis was completed for GLP-1 users vs diabetic non-GLP users to control for the large percentage of diabetic patients in the GLP-1 user population. This analysis preserved sample size and statistical power, minimizing the risk of type II statistical error, while isolating the GLP-1 group as a whole compared to a diabetic cohort. This balancing was completed using a prior clinical diagnosis of diabetes or an HbA1c value of >6.5%.

Nutritional and laboratory data were obtained from preoperative labs and included HbA1c, hemoglobin (sex-adjusted), serum albumin, total protein, prealbumin, calcium, alkaline phosphatase, and 25-hydroxy vitamin D (25[OH]D). These labs were drawn within 2-4 weeks of operative intervention. Malnutrition was defined as the presence of at least 1 laboratory value below the institutional lower limit of normal. Severe malnutrition was defined as 2 or more nutritional deficiencies. The presence of prescribed vitamin D supplementation and its dosage were also recorded. HbA1c, with levels <6.5% were considered controlled. HbA1c being elevated >6.5% was not considered to be a marker of malnutrition, however. Preoperative nutrition labs are obtained routinely rather than selectively in the authors’ practice. Abnormal results did not prompt surgical delay, but they were addressed with counseling and nutritional supplementation was encouraged perioperatively. Unlike conditions such as poorly controlled diabetes, there is limited evidence to support delaying arthroplasty for malnutrition, though it may theoretically improve outcomes.

Laboratory reference ranges were defined based on standard laboratory norms. Hemoglobin reference values were stratified by sex: 13.7-17.5 g/dL for men and 11.5-14.9 g/dL for women. Normal serum albumin was defined as 3.5-5.0 g/dL, and total protein as 6.2-8.1 g/dL. Prealbumin levels were considered normal between 17-34 mg/dL. Serum calcium reference range was 8.4-10.2 mg/dL, alkaline phosphatase 40-150 IU/L, and vitamin D-25 Hydroxy serum levels were considered normal between 6.6-49.9 ng/mL.

GLP-1 use was documented, including the primary indication and length of use in months.

### Statistical analysis

Descriptive statistics were used to summarize patient demographics, clinical characteristics, and laboratory values. Continuous variables were reported as means with standard deviations. Categorical variables were expressed as frequencies and percentages. Patients were stratified based on nutritional status, and comparisons between groups were performed using unpaired 2-tailed Student’s t-tests for continuous variables and chi-square or Fisher’s exact tests for categorical variables. A *P* value < .05 was considered statistically significant. Odds ratios were calculated utilizing Fisher Exact Testing for final malnourishment evaluation.

A post hoc power analysis was conducted to determine the minimum sample size required to detect a difference in malnutrition prevalence between the 2 groups. We assumed malnutrition rates of 8.5% in non-GLP-1 patients and 35% in GLP-1 patients, with a 2-sided α of 0.05 and power of 80% (β = 0.20), the calculated sample size was at least 29 patients per group. These assumptions were based on prior arthroplasty and nutritional literature with a bias toward assuming a smaller difference between groups to avoid a type 2 statistical error [11,[17], [18], [19]]. All statistical analyses were performed using Microsoft Excel.

## Results

A total of 165 patients who underwent elective TJA were included in the final analysis. Of these, 29 patients (17.6%) were actively using GLP-1 receptor agonists at the time of surgery, while 136 patients (82.4%) were not. The GLP-1 group comprised 86% TKA and 14% THA, compared to 65% TKA and 35% THA in the non-GLP-1 group.

The mean age at surgery was 63.8 ± 9.4 years in the GLP-1 group and 64.3 ± 10.9 years in the non-GLP-1 group. 75.8% of GLP-1 users and 55.9% of nonusers were women. The overall cohort consisted of patients primarily from white or Hispanic backgrounds. The mean BMI was significantly higher in the GLP-1 group 35.6 ± 6.1 kg/m^2^ compared to nonusers 31.4 ± 6.3 kg/m^2^, *P* = .001 (Table 1).

**Table 1:** Population demographics and clinical data.

Characteristic	GLP-1 agonist nonusers	GLP-1 agonist users
Female, n (%)	76 (55.9%)	22 (75.8%)
Age, y (median, SD)	64.3 (10.9)	63.8 (9.4)
BMI, kg/m^2^ (median, SD)	31.38 (6.3)	35.6 (6.1)
Substance use, n (%)		
Smoking history	37 (27.2%)	13 (44.8%)
Recreational drug use	2 (1.47%)	2 (6.9%)
Alcohol use	60 (44.1%)	9 (31%)
Procedure performed, n (%)		
TKA	89 (65.4%)	25 (86.2%)
THA	47 (34.6%)	4 (13.8%)
Comorbidities, n (%)		
Hypertension	81 (59.6%)	25 (86.2%)
Diabetes mellitus	22 (16.2%)	22 (75.8%)
Hyperlipidemia	48 (35.3%)	17 (58.6%)
Coronary artery disease	8 (5.9%)	2 (6.9%)
CCI (median, standard deviation)	2.21 (1.35)	2.72 (1.1) *P = .055*

Comorbidities were common across both groups with hypertension, diabetes mellitus, hyperlipidemia, and prior tobacco use being the most common. CCI score for the GLP-1 group was 2.72 ± 1.1, and the non-GLP-1 group was 2.21 ± 1.35 (*P* = .055).

GLP-1 agonists were prescribed for diabetes (8 patients), weight loss (9 patients), or both (12 patients), with a median duration of use of 9.6 months.

**Figure 1:**
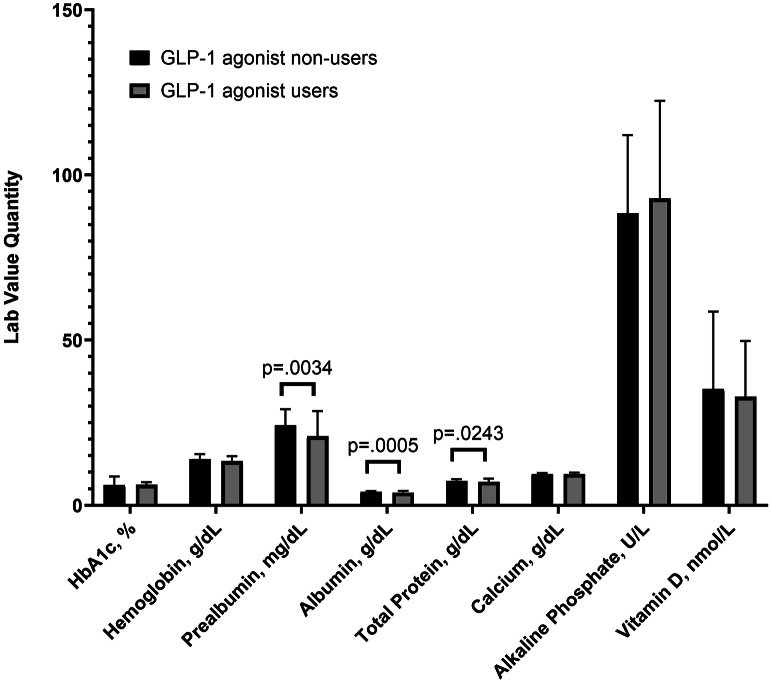
Mean nutritional lab values for each group.

The mean HbA1c level was 6.17% ± 0.80 in the GLP-1 group and 6.07% ± 2.65 in the non-GLP-1 group (*P* = .841). Hemoglobin levels averaged 13.39 ± 1.52 g/dL in GLP-1 users and 13.95 ± 1.54 g/dL in nonusers (*P* = .075). Albumin concentrations were 3.77 ± 0.58 g/dL in GLP-1 users and 4.02 ± 0.28 g/dL in nonusers (*P* < .001). Total protein levels were 7.08 ± 0.99 g/dL in the GLP-1 group and 7.37 ± 0.52 g/dL in the non-GLP-1 group (*P* = .024). Prealbumin values were 20.93 ± 7.59 mg/dL in GLP-1 users and 24.24 ± 4.86 mg/dL in nonusers (*P* = .003). All protein laboratory measurements were statistically significant between groups. Calcium levels were measured at 9.41 ± 0.51 mg/dL for GLP-1 users and 9.49 ± 0.34 mg/dL for nonusers (*P* = .197). Alkaline phosphatase values were 92.86 ± 29.56 IU/L and 88.37 ± 23.65 IU/L in GLP-1 and non-GLP-1 groups, respectively (*P* = .550). 25-hydroxy vitamin D levels averaged 32.86 ± 16.89 ng/mL in GLP-1 users and 35.21 ± 23.36 ng/mL in nonusers (*P* = .239) (Table 2). This information can be found in Figure 1.

**Table 2:** Population nutritional lab markers.

Characteristic	GLP-1 agonist nonusers	GLP-1 agonist users	*P* value
HbA1c, % (median, SD)	6.07 (2.65)	6.17 (0.8)	.8409
Hemoglobin, g/dL (median, SD)	13.95 (1.54)	13.39 (1.52)	.0748
Prealbumin, mg/dL (median, SD)	**24.24 (4.86)**	**20.93 (7.59)**	**.0034**
Albumin, g/dL (median, SD)	**4.02 (0.28)**	**3.77 (0.58)**	**.0005**
Total Protein, g/dL (median, SD)	**7.37 (0.52)**	**7.08 (0.99)**	**.0243**
Calcium, g/dL (median, SD)	9.49 (0.34)	9.41 (0.51)	.1970
Alkaline Phosphate, U/L (median, SD)	88.37 (23.7)	92.86 (29.6)	.5504
Vitamin D, nmol/L (median, SD)	35.21 (23.4)	32.86 (16.9)	.2392
Malnourished state, n (%)	**12 (8.8%)**	**11 (38%)**	**.0003**
Severely malnourished state, n (%)	**4 (2.9%)**	**5 (17.2%)**	**.0091**

Bolding indicates statistical significance.

**Figure 2:**
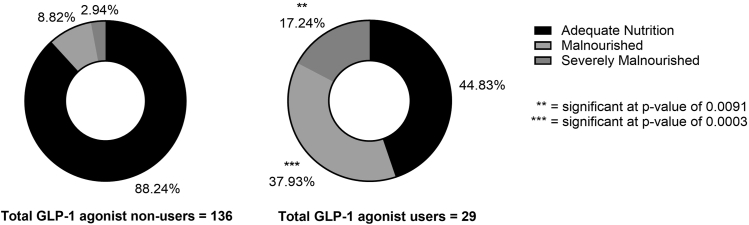
Percentage of patients in each group that are nourished, malnourished, or severely malnourished based on laboratory data.

**Figure 3:**
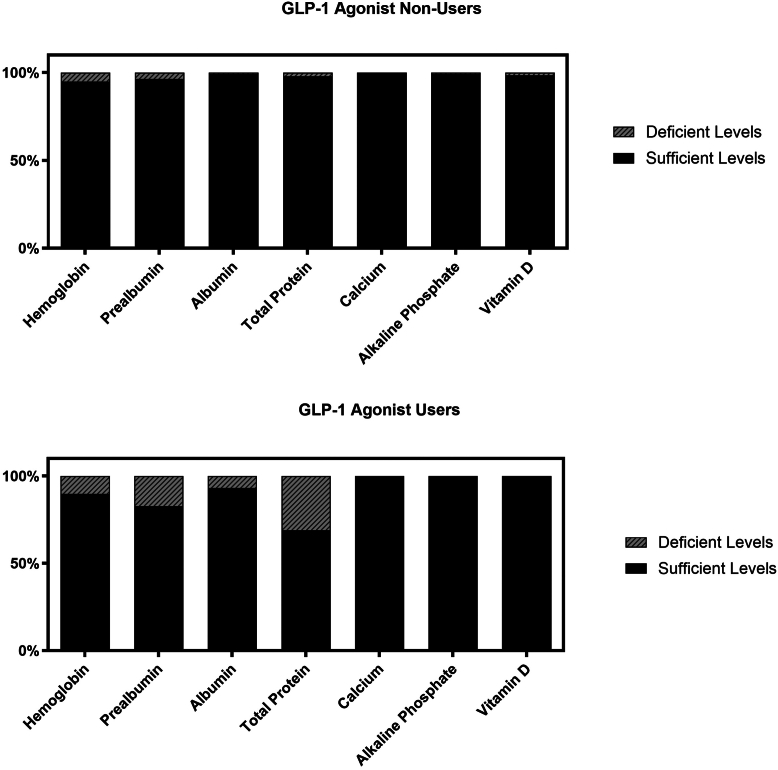
Percentage of nutritional lab values that are deficient in each group. Deficient is defined as at least 1 deficient lab value.

Malnutrition, defined by the presence of at least 1 abnormal nutritional lab value, was observed in 11 **(38%)** of GLP-1 users and 12 **(8.8%)** of nonusers (odds ratio [OR] = 6.2, *P* = <0.001). Severe malnutrition (≥2 lab deficiencies) was identified in 5 **(17.2%)** of GLP-1 users vs **4 (2.9%)** of nonusers (OR = 6.88, *P* = .009) (Fig. 2). The number of malnourished patients with deficient hemoglobin levels was 3 (10.3%) in the GLP-1 group and 7 (5.1%) in the non-GLP-1 group. Prealbumin deficiency was observed in 5 (17.2%) GLP-1 users compared to 5 (3.7%) nonusers. Low albumin was found in 2 (6.9%) GLP-1 patients and 1 (0.73%) nonuser, while total protein deficiency was present in 9 (31%) GLP-1 users vs 3 (2.2%) nonusers. Alkaline phosphatase abnormalities occurred in 0 GLP-1 users and 1 (0.73%) nonuser (Fig. 3). Vitamin D deficiency was observed only in 2 (1.5%) non-GLP-1 users, and no calcium abnormalities were reported in either group (Table 3).

**Table 3:** Malnourished and severely malnourished patients with deficient lab values.

Lab value	Deficient GLP-1 agonist nonusers, n (%)	Deficient GLP-1 agonist users, n (%)	*P* value
Hemoglobin	7 (5.1%)	3 (10.3%)	-
Prealbumin	5 (3.7%)	5 (17.2%)	-
Albumin	1 (0.73%)	2 (6.9%)	-
Total protein	3 (2.2%)	9 (31%)	-
Calcium	0 (0%)	0 (0%)	-
Alkaline phosphate	1 (0.73%)	0 (0%)	-
Vitamin D	2 (1.5%)	0 (0%)	-
Malnourished state	**12 (8.8%)**	**11 (38%)**	**.0003**
Severely malnourished state	**4 (2.9%)**	**5 (17.2%)**	**.0091**

Bolding indicates statistical significance.

The subgroup analysis controlling for diabetes and HbA1c had 26 non-GLP-1 users. This group was older (*P* = .07), approaching significance, had a higher percentage of diabetic patients 85%, had a significantly higher comorbidity index, 3.57 vs 2.72 (*P* = .014), and had a significantly higher mean HbA1c, 6.69 vs 6.16 (*P* = .01). Despite this non-GLP-1 group being objectively more unhealthy, the GLP-1 cohort still had a significantly higher odds of being malnourished (OR = 4.69, *P* = .032).

## Discussion

GLP-1 agonist use has exponentially increased over the past decade and has become an attractive treatment option for weight management. Because of this, patients who may not have historically been candidates for TJA are now meeting the BMI goals required to undergo these elective surgeries. Recently, review studies and large database studies have supported the judicious use of GLP-1 agonists to potentially improve outcomes and reduce complication rates [[20], [21], [22]]. Although database studies provide large patient volume, it is difficult to control for confounding variables, making conclusions difficult to extrapolate. Additionally, no study to date has evaluated or compared the preoperative nutritional status and protein reserves of GLP-1 TJA patients and compared those values to a non-GLP-1 control cohort. This study found that patients taking GLP-1 agonists preoperatively had approximately 7 times the odds of being severely malnourished compared to non-GLP-1 patients (*P* < .001) (Fig. 2). Even when controlling for diabetes via a subcohort analysis, the GLP-1 group still had approximately 5 times the odds of being malnourished compared to non-GLP-1 patients, despite non-GLP-1 users being objectively more unwell based on other comorbid conditions, HbA1c levels, and age (*P* = .01). The GLP-1 group was also found to have significantly lower protein reserves in all 3 protein measurements, including prealbumin (*P* = .003), albumin (*P* < .001), and total protein (*P* = .02) (Fig. 1). As arthroplasty surgeons continue to holistically evaluate TJA patients, the association between GLP-1 use and malnutrition should be examined with increased scrutiny.

Preoperative evaluation and optimization in TJA has become the standard of care for most high-volume arthroplasty surgeons [23]. Debate still exists, however, on how to efficiently and effectively identify and manage comorbidities to optimize these patients for surgery [24,25]. Multiple protocols have surfaced, each requiring substantial monetary and health professional time resources to properly execute [[26], [27], [28]]. With recent increased focus on health-care spending, it is imperative to identify which TJA patients need specialized nutritional assessment and preoperative supplementation and optimization for proper allocation of resources. Similar literature can be found on postbariatric surgery patients, where the standard of care is to delay TJA for up to 1 year to allow for preoperative nutritional evaluation in this highly malnourished group [29]. GLP-1 users in this study were at substantial risk of malnutrition, with over 40% of the population having at least 1 baseline nutritional deficit. Recognizing that GLP-1 users are at high risk for malnutrition allows for pre-emptive focusing of attention and resources on this patient subset, optimizing nutritional status and potentially improving outcomes and complication rates.

### Limitations

This study has several limitations. It was conducted at a single institution in a large urban setting, which may limit the generalizability to other populations or practice environments. Additionally, although the current findings provide valuable insights, long-term outcomes remain unknown outside of large database studies which present their own limitations and biases. Future work should include expanding this cohort through ongoing data collection and potentially multi-institution collaboration to enable prospective or larger-scale retrospective analyses. Target areas of investigation include differentiating active weight loss vs plateau status, assessing the impact of targeted nutritional supplementation perioperatively, comparing prescription indications, and evaluating both short- and long-term postoperative outcomes. This work would greatly expound on the current topic and help guide management of this unique patient population.

## Conclusions

This study evaluated the preoperative nutritional status of patients undergoing primary TJA, with a focus on those using GLP-1 receptor agonists for weight loss or diabetes management. While these medications have shown promise in reducing weight and improving surgical candidacy, these findings suggest that GLP-1 users are at substantially increased risk for nutritional deficiencies prior to surgery. The study highlights the importance of careful nutritional assessment and optimization in this growing patient population. As GLP-1 use expands, targeted preoperative evaluation may help reduce complications and improve outcomes following arthroplasty procedures.

## CRediT authorship contribution statement

**Zachary Jodoin:** Writing – review & editing, Writing – original draft, Supervision, Investigation, Formal analysis, Data curation, Conceptualization. **William H. Young:** Writing – review & editing, Writing – original draft, Conceptualization. **Daanish Sheikh:** Visualization, Data curation. **Belinda Pena:** Data curation. **Chance C. Moore:** Writing – review & editing, Supervision. **Frank Buttacavoli:** Writing – review & editing, Supervision.

## Conflicts of interest

Frank Buttacavoli is on the speakers' bureau/paid presentations for Zimmer Biomet, Sanara Medtech, Solventum, Heraus, and Medtronic and is a paid consultant for Zimmer Biomet, Sanara Medtech, Solventum, Heraus, and Medtronic.

The other authors declare no potential conflicts of interest.

For full disclosure statements refer to https://doi.org/10.1016/j.artd.2025.101865.

## Appendix A Supplementary data

Conflict of Interest Statement for Pena

Conflict of Interest Statement for Moore

Conflict of Interest Statement for Sheikh

Conflict of Interest Statement for Buttacavoli

Conflict of Interest Statement for Young

Conflict of Interest Statement for Jodoin

## References

[1] Kerkhoffs G.M., Servien E., Dunn W., Dahm D., Bramer J.A., Haverkamp D. (2012). The influence of obesity on the complication rate and outcome of total knee arthroplasty: a meta-analysis and systematic literature review. J Bone Joint Surg Am.

[2] Onggo J.R., Ang J.J.M., Onggo J.D., de Steiger R., Hau R. (2021). Greater risk of all-cause revisions and complications for obese patients in 3 106 381 total knee arthroplasties: a meta-analysis and systematic review. ANZ J Surg.

[3] Jung P., Morris A.J., Zhu M., Roberts S.A., Frampton C., Young S.W. (2017). BMI is a key risk factor for early periprosthetic joint infection following total hip and knee arthroplasty. N Z Med J.

[4] National Institute of Diabetes and Digestive and Kidney Diseases (2021). Overweight & obesity statistics. National Institute of Diabetes and Digestive and Kidney Diseases. https://www.niddk.nih.gov/health-information/health-statistics/overweight-obesity.

[5] Gupta A., Jelinek H.F., Al-Aubaidy H. (2017). Glucagon like peptide-1 and its receptor agonists: their roles in management of type 2 diabetes mellitus. Diabetes Metab Syndr.

[6] George C., Byun A., Howard-Thompson A. (2018). New injectable agents for the treatment of type 2 diabetes part 2-Glucagon-Like Peptide-1 (GLP-1) agonists. Am J Med.

[7] Sandsdal R.M., Juhl C.R., Jensen S.B.K., Lundgren J.R., Janus C., Blond M.B. (2023). Combination of exercise and GLP-1 receptor agonist treatment reduces severity of metabolic syndrome, abdominal obesity, and inflammation: a randomized controlled trial. Cardiovasc Diabetol.

[8] White G.E., Shu I., Rometo D., Arnold J., Korytkowski M., Luo J. (2023). Real-world weight-loss effectiveness of glucagon-like peptide-1 agonists among patients with type 2 diabetes: a retrospective cohort study. Obesity (Silver Spring).

[9] Vilsbøll T., Christensen M., Junker A.E., Knop F.K., Gluud L.L. (2012). Effects of glucagon-like peptide-1 receptor agonists on weight loss: systematic review and meta-analyses of randomised controlled trials. BMJ.

[10] Mahase E. (2024). GLP-1 agonists: US sees 700% increase over four years in number of patients without diabetes starting treatment. BMJ.

[11] Eminovic S., Vincze G., Eglseer D., Riedl R., Sadoghi P., Leithner A. (2021). Malnutrition as predictor of poor outcome after total hip arthroplasty. Int Orthop.

[12] Nickel B.T., Klement M.R., Penrose C.T., Green C.L., Seyler T.M., Bolognesi M.P. (2016). Lingering risk: bariatric surgery before total knee arthroplasty. J Arthroplasty.

[13] Liu J.X., Paoli A.R., Mahure S.A., Bosco J., Campbell K.A. (2020). Preoperative bariatric surgery utilization is associated with increased 90-day postoperative complication rates after total joint arthroplasty. J Am Acad Orthop Surg.

[14] Smith T.O., Aboelmagd T., Hing C.B., MacGregor A. (2016). Does bariatric surgery prior to total hip or knee arthroplasty reduce post-operative complications and improve clinical outcomes for Obese patients?. Bone Joint J.

[15] Sax O.C., Chen Z., Bains S.S., Salib C.G., Pervaiz S.S., Mont M.A. (2022). Timing and type of bariatric surgery preceding total knee arthroplasty leads to similar complications and outcomes. J Arthroplasty.

[16] Sergi G., Coin A., Enzi G. (2006). Role of visceral proteins in detecting malnutrition in the elderly. Eur J Clin Nutr.

[17] Phillips J.L.H., Ennis H.E., Jennings J.M., Dennis D.A. (2023). Screening and management of malnutrition in total joint arthroplasty. J Am Acad Orthop Surg.

[18] Golladay G.J., Satpathy J., Jiranek W.A. (2016). Patient optimization—strategies that work: malnutrition. J Arthroplasty.

[19] Morey V.M., Song Y.D., Whang J.S., Kang Y.G., Kim T.K. (2016). Can serum albumin level and total lymphocyte count be surrogates for malnutrition to predict wound complications after total knee arthroplasty?. J Arthroplasty.

[20] Jacofsky D.J., Springer B.D., Mont M.A., Ushakumari D.S., Sladen R.N. (2024). The impact of glucagon-like Peptide-1 agonists on hip and knee arthroplasty and perioperative considerations. J Arthroplasty.

[21] Kim B.I., LaValva S.M., Parks M.L., Sculco P.K., Della Valle A.G., Lee G.C. (2025). Glucagon-like Peptide-1 receptor agonists decrease medical and surgical complications in morbidly Obese patients undergoing primary TKA. J Bone Joint Surg Am.

[22] Kim B.I., Khilnani T.K., LaValva S.M., Goodman S.M., Della Valle A.G., Lee G.C. (2024). Utilization of glucagon-like Peptide-1 receptor agonist at the time of total hip arthroplasty for patients who have morbid obesity. J Arthroplasty.

[23] Johns W.L., Layon D., Golladay G.J., Kates S.L., Scott M., Patel N.K. (2020). Preoperative risk factor screening protocols in total joint arthroplasty: a systematic review. J Arthroplasty.

[24] Berend K.R., Lombardi A.V., Mallory T.H. (2004). Rapid recovery protocol for peri-operative care of total hip and total knee arthroplasty patients. Surg Technol Int.

[25] Buchanan M.W., Gibbs B., Ronald A.A., Novikov D., Yang A., Salavati S. (2024). Is a rapid recovery protocol for THA and TKA associated with decreased 90-day complications, opioid use, and readmissions in a health safety-net Hospital?. Clin Orthop Relat Res.

[26] Dlott C.C., Moore A., Nelson C., Stone D., Xu Y., Morris J.C. (2020). Preoperative risk factor optimization lowers hospital length of stay and postoperative emergency department visits in primary total hip and knee arthroplasty patients. J Arthroplasty.

[27] Turcotte J., Menon N., Angeles J., Zaidi A., King P., MacDonald J. (2021). A rapid recovery protocol applied to total joint arthroplasty reduced readmissions for surgical but not medical reasons over a 5-Year period. HSS J.

[28] Taylor A.J., Kay R.D., Bryman J.A., Tye E.Y., Longjohn D.B., Najibi S. (2022). Outcomes of an institutional rapid recovery protocol for total joint arthroplasty at a safety net hospital. J Am Acad Orthop Surg Glob Res Rev.

[29] Schwarzkopf R., Lavery J.A., Hooper J., Parikh M., Gold H.T. (2018). Bariatric surgery and time to total joint arthroplasty: does it affect readmission and complication rates?. Obes Surg.

