# Supplementary material for: Malnutrition is Common in Patients Utilizing Glucagon-Like Peptide-1 Agonists Prior to Total Joint Arthroplasty
Source: Arthroplast Today. 2025 Sep 30;35:101865. doi: 10.1016/j.artd.2025.101865 (PMC12512160; doi:10.1016/j.artd.2025.101865)

**CONFLICT OF INTEREST STATEMENT**

***American Association of Hip and Knee Surgeons***

(Adopted from the American Academy of Orthopaedic Surgeons disclosure statement)

The following form **must be filled out completely and submitted by each author (example, 6 authors, 6 forms).**

**All items require a response. If there is no relevant disclosure for a given item, enter "*None*.”**

Manuscript Title

1. Royalties from a company or supplier (The following conflicts were disclosed)

none

1. Speakers bureau/paid presentations for a company or supplier (The following conflicts were disclosed)

**Zimmer biomet, sanara medtech, solventum, heraus, medtronic**

3A. Paid employee for a company or supplier (The following conflicts were disclosed)

none

3B. Paid consultant for a company or supplier (The following conflicts were disclosed)

**Zimmer biomet, sanara medtech, solventum, heraus, medtronic**

3C. Unpaid consultants for a company or supplier (The following conflicts were disclosed)

none

1. Stock or stock options in a company or supplier (The following conflicts were disclosed)

none

1. Research support from a company or supplier as a Principal Investigator (The following conflicts were disclosed)

None in last 2 years

1. Other financial or material support from a company or supplier (The following conflicts were disclosed)

none

1. Royalties, financial or material support from publishers (The following conflicts were disclosed)

none

1. Medical/Orthopaedic publications editorial/governing board (The following conflicts were disclosed)

none

1. Board member/committee appointments for a society (The following conflicts were disclosed)

Aaos hip knee evaluation committee

**Each author must sign AND print or type his/her name, date and submit a separate form**

In addition, one BLINDED Conflict of Interest form (no author names used) should be submitted per manuscript with all author disclosures.

Frank Buttacavoli 4/20/2025


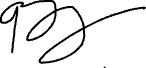


Author Name (Print or Type) Author Signature Date


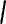

Supplement: Conflict of Interest Statement for Buttacavoli [file mmc4.docx]
